# Desmoid Fibromatosis Involving the Retropharyngeal and Danger Spaces: A Case Report

**DOI:** 10.7759/cureus.75969

**Published:** 2024-12-18

**Authors:** Masami Ito, Kota Yokoyama, Yosuke Ariizumi, Yoshihito Kano, Kurara Yamamoto, Takahiro Asakage, Ukihide Tateishi

**Affiliations:** 1 Diagnostic Radiology and Nuclear Medicine, Institute of Science Tokyo, Tokyo, JPN; 2 Head and Neck Surgery, Institute of Science Tokyo, Tokyo, JPN; 3 Clinical Oncology, Institute of Science Tokyo, Tokyo, JPN; 4 Human Pathology, Institute of Science Tokyo, Tokyo, JPN

**Keywords:** desmoid fibromatosis, magnetic resonance imaging, positron emission tomography, radiotherapy, retropharyngeal space

## Abstract

Desmoid fibromatosis (DF) is a rare, non-metastasizing but locally aggressive mesenchymal tumor arising from fibroblasts or myofibroblasts. We report a solitary case of DF involving the retropharyngeal and danger spaces, a location rarely documented. The patient, a woman in her 70s, presented with progressive pharyngeal discomfort over six months. Imaging studies revealed a soft-tissue mass consistent with DF, and the diagnosis was confirmed pathologically. The tumor demonstrated unique imaging findings, including band-like hypointense areas on T2-weighted imaging on magnetic resonance imaging and moderate 2-deoxy-2-[^18^F]fluoro-D-glucose uptake on positron emission tomography/computed tomography (PET/CT), which may aid in differentiating DF from malignant tumors in atypical locations. Furthermore, this case highlights the effectiveness of radiotherapy in achieving significant tumor reduction, offering insights into the management of DF in rare and challenging locations. These findings not only aid in refining the differential diagnosis but also provide evidence supporting radiotherapy as a viable treatment option in cases where surgical resection is unfeasible.

## Introduction

Desmoid fibromatosis (DF), also known as aggressive fibromatosis or desmoid tumor, is a rare, deep-seated neoplasm arising from fibroblasts or myofibroblasts [1]. It is characterized by locally aggressive, infiltrative growth and a propensity for local recurrence, but it does not metastasize. The estimated incidence is less than four cases per million population per year [2]. While traditionally cited as affecting one in 35,000 individuals and occurring twice as often in females as in males [3], more recent epidemiological data suggest a median age of 37-39 years at diagnosis, with a female predominance (M:F ratio of 0.5:1), though this sex distribution evens out in pediatric and post-childbearing-age patients [4,5].

The etiology of DF is multifactorial, involving both genetic and physical factors. The majority (90%-95%) of sporadic cases are associated with somatic mutations in the CTNNB1 gene, particularly at codons 41 and 45 of exon 3 (encoding β-catenin), with p.Thr41Ala and p.Ser45Phe being the most common mutations [1,3,6]. Conversely, hereditary cases, often associated with familial adenomatous polyposis (FAP) or Gardner syndrome, are linked to germline APC gene mutations [6]. Physical factors such as trauma, prior surgery, and pregnancy have also been implicated [7]. While MRI is the preferred imaging modality, showing mixed hyperintense/isointense T2 signals [8], 2-deoxy-2-[^18^F]fluoro-D-glucose (FDG) positron emission tomography-computed tomography (PET/CT) interpretation in DF can be challenging. DF, especially active lesions, can demonstrate significant FDG uptake, mimicking malignancy and leading to misdiagnosis, as highlighted in several studies [9,10].

Standard treatment ranges from active surveillance for asymptomatic lesions to surgery, radiotherapy, and systemic therapies such as nonsteroidal anti-inflammatory drugs (NSAIDs), anti-estrogens, and tyrosine kinase inhibitors [1].

This report describes a rare case of extra-abdominal DF in the retropharyngeal and danger spaces, where initial moderate FDG avidity raised suspicion for malignancy. This case emphasizes the diagnostic difficulty posed by DF in unusual locations with moderate FDG avidity, while also demonstrating a successful treatment outcome. We aim to highlight the importance of considering DF in the differential diagnosis of moderately FDG-avid lesions in rare locations, especially when characteristic MRI findings are present.

## Case presentation

A woman in her 70s, with no significant medical history other than an appendectomy at age 20, presented with a six-month history of progressive pharyngeal discomfort. At her initial hospital visit, physical examination revealed swelling of the posterior pharyngeal mucosa, and endoscopic evaluation confirmed mucosal thickening of the posterior pharyngeal wall. A biopsy under ultrasound guidance identified features consistent with a spindle cell tumor, but as a definitive diagnosis could not be established, the patient was referred to our hospital for specialized evaluation and treatment. She had no significant family history, including familial adenomatous polyposis, and her laboratory findings were unremarkable.

**Figure 1:**
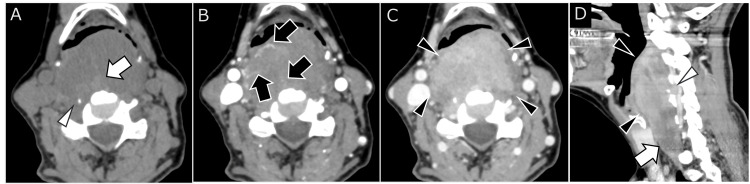
Non-contrast and contrast-enhanced CT images. A soft-tissue mass extends from the retropharyngeal to the danger space at the oropharynx to the thyroid level. (A) Non-contrast CT: The majority of the mass is hypodense, with streak-like hyperdense areas (white arrow) and microcalcifications (arrowhead). (B) Early-phase contrast-enhanced CT: Linear early enhancement is observed at the periphery and within the tumor (black arrows). (C) Delayed-phase contrast-enhanced CT: Most of the remaining mass shows delayed enhancement (black arrowheads), which is suspected to correspond to proliferative spindle cells and collagenous stroma. (D) Delayed-phase contrast-enhanced CT, sagittal view: Most of the tumor exhibits enhancement, suggesting fibrous components (black arrowhead). However, the caudal region shows hypoattenuation, suspected to correspond to myxoid stroma (white arrow). Protrusion into the right C4/5 intervertebral foramen is observed, with posterior displacement of the right vertebral artery (white arrowhead).

CT revealed a 50 × 39 × 81 mm soft-tissue mass involving the retropharyngeal and danger spaces, extending along the longus colli muscle. The tumor showed predominantly low attenuation with streak-like hyperattenuating areas. Delayed enhancement was observed, corresponding to spindle cell proliferation and collagenous stroma (Figure 1).

**Figure 2:**
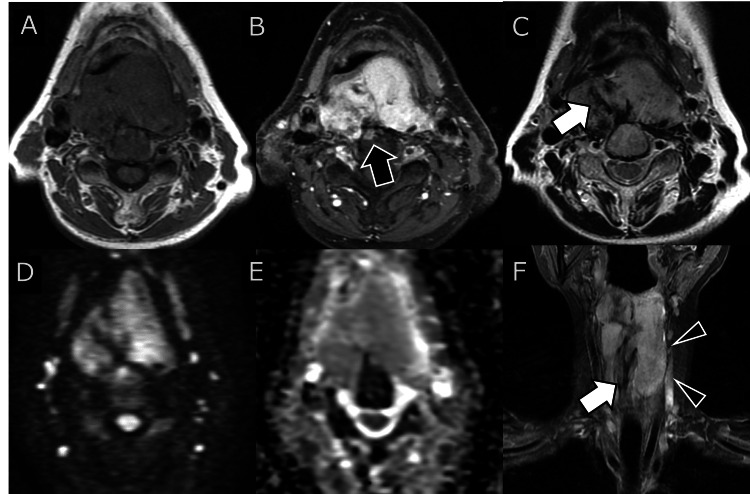
Magnetic resonance imaging (MRI) findings of the lesion. (A) T1-weighted image: The tumor demonstrates signal intensity comparable to or slightly lower than that of muscle. (B) Contrast-enhanced T1WI with fat suppression: The tumor is uniformly enhanced, with suspected invasion into the C4 vertebral body (black arrow). (C) T2-weighted image: Hyperdense regions on CT appear as band-like hypointense areas (white arrow), suggesting flow voids, hemosiderin deposition, or calcifications. (D) Diffusion-weighted imaging: The solid component exhibits uniform, mildly high signal intensity. (E) Apparent diffusion coefficient (ADC) map: The ADC value shows mild reduction without pronounced decrease. (F) Short tau inversion recovery, coronal view: The solid component of the tumor exhibits relatively uniform signal intensity with capsular-like structures (black arrowheads) and clear demarcation from surrounding tissues. Band-like hypointense areas are visible in the central portion (white arrow).

Magnetic resonance imaging (MRI) findings included iso- to mildly hyperintense signals on T1-weighted images (T1WI) and heterogeneous, predominantly hyperintense signals on T2-weighted images (T2WI), with band-like hypointense regions suggestive of dense collagenous stroma. Diffusion-weighted imaging demonstrated high signal intensity without a marked decrease in the apparent diffusion coefficient (Figure 2).

**Figure 3:**
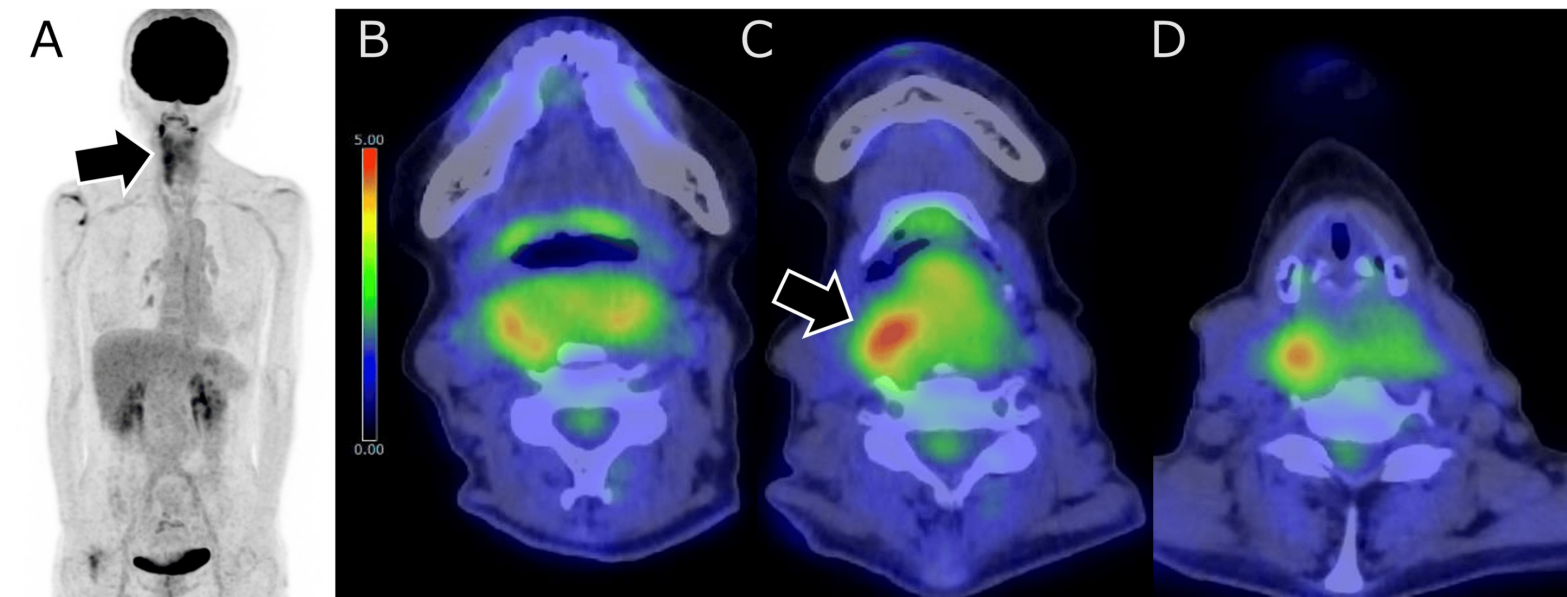
FDG PET/CT findings of the whole body and the lesion. (A) The maximum intensity projection image shows high FDG uptake in the cervical region (black arrow), with no abnormal uptake detected elsewhere in the body. (B-D) Fusion images of FDG PET and CT reveal moderate FDG uptake (SUVmax 5.3) in the solid component of the tumor, with the highest uptake indicated by the black arrow. In contrast, the caudal portion, which demonstrates low attenuation and poor enhancement on CT, shows minimal FDG uptake comparable to background activity. FDG, 2-deoxy-2-[18F]fluoro-D-glucose; PET, positron emission tomography; CT, computed tomography

On FDG PET/CT, moderate FDG uptake with a maximum standardized uptake value of 5.3 was observed in the solid component, while the less-enhancing portions exhibited low uptake. No distant metastases were identified (Figure 3).

The imaging findings suggested a rapidly growing tumor with both fibrous and myxoid components, and the detection of spindle cells in the biopsy prompted consideration of several differential diagnoses. Synovial sarcoma, known for its potential to arise in the retropharyngeal space, was considered due to its rapid progression. Malignant peripheral nerve sheath tumor, which can occur sporadically despite its common association with neurofibromatosis, was another possibility given the high FDG uptake and its frequent occurrence in similar anatomical locations [11,12]. While ektomesenchymoma has been reported as a differential diagnosis in this region, it was excluded in this case due to the patient's age being inconsistent with typical presentations [12]. Additionally, a solitary fibrous tumor was considered based on its spindle cell morphology, internal vascular structures, and the characteristic mix of T2-hyperintense and T2-hypointense components, commonly referred to as the *yin-yang sign*, which supported its inclusion in the differential diagnosis [13]. Given the potential malignancy and the risk of airway obstruction, early diagnosis and treatment were deemed necessary, including a transoral biopsy performed under general anesthesia.

**Figure 4:**
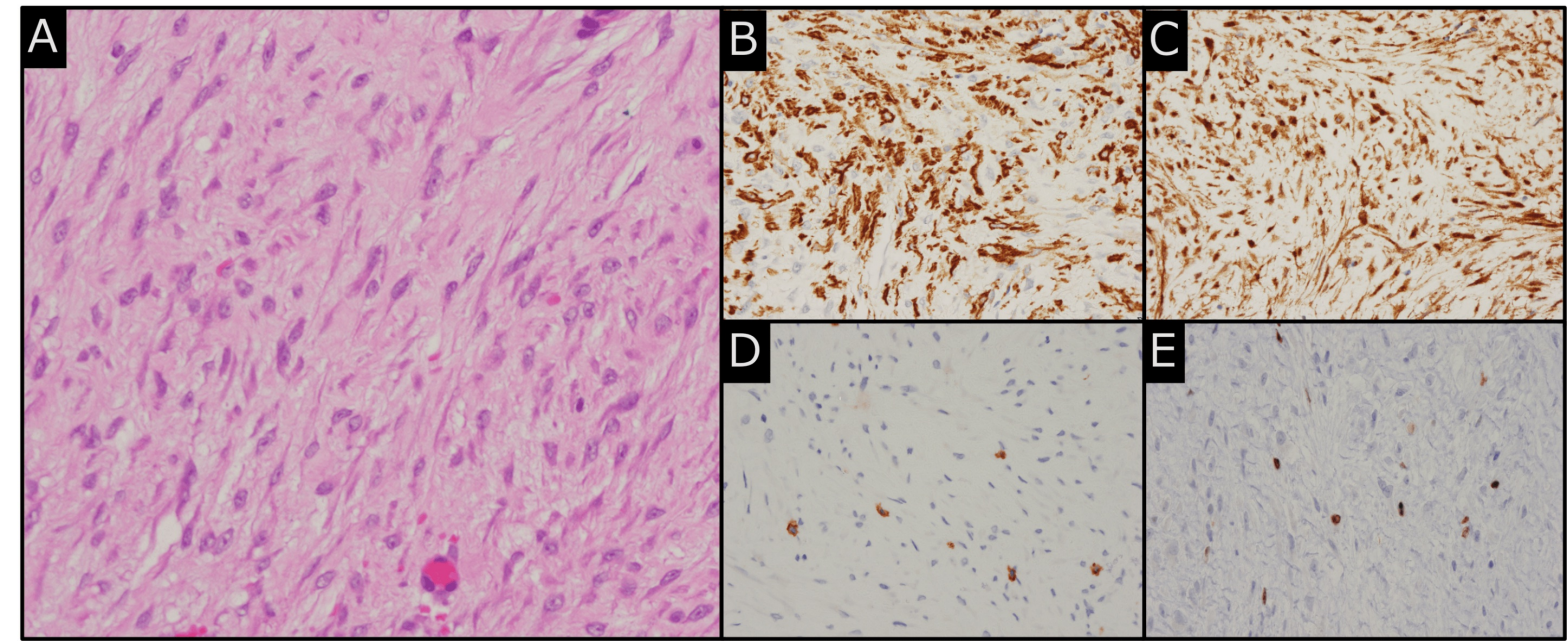
Histopathological findings of the biopsy specimens. (A) Hematoxylin and eosin (H&E) staining: Proliferation of spindle-shaped cells with eosinophilic cytoplasm and oval to polygonal nuclei. Mild nuclear atypia and rare mitotic figures are observed. (B) Immunohistochemistry (IHC) for α-smooth muscle actin (SMA): Positive staining in the tumor cells. (C) IHC for β-catenin: Strong positive nuclear staining in the tumor cells, a key feature of desmoid fibromatosis. (D) IHC for c-kit: Weak focal staining was observed; however, this was nonspecific. CD34 and DOG-1 staining were negative, effectively ruling out spindle cell gastrointestinal stromal tumor as a differential diagnosis. (E) IHC for Ki67: The proliferation index was 1.67%, indicating a low mitotic rate.

Pathological examination of the tumor revealed spindle-shaped cells with eosinophilic cytoplasm and oval to polygonal nuclei with mild anisokaryosis. Rare mitotic figures (1/10 HPF) were noted. Immunohistochemical analysis showed nuclear β-catenin positivity and α-smooth muscle actin positivity, with negative staining for markers such as S-100, CD34, Desmin, discovered on gastrointestinal stromal tumor-1 (DOG-1), anaplastic lymphoma kinase (ALK), and signal transducer and activator of transcription 6 (STAT6). Genetic testing did not detect *APC* gene mutations, leading to a final diagnosis of sporadic DF (Figure 4).

**Figure 5:**
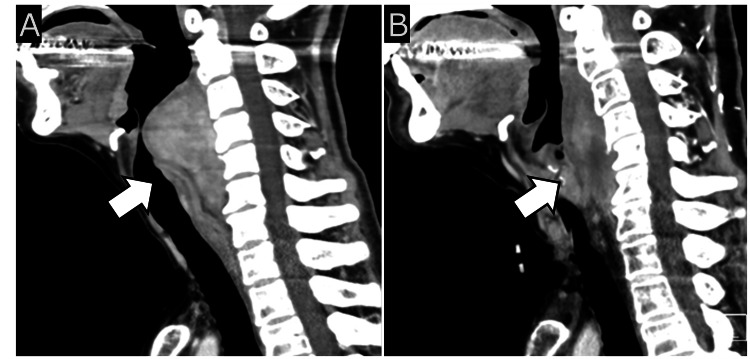
Pre- and post-treatment CT findings. (A) Pre-treatment CT: Sagittal contrast-enhanced CT image showing a large, irregularly enhanced mass (white arrow) occupying the retropharyngeal and danger spaces, extending to the thyroid level. (B) Post-treatment CT: Sagittal contrast-enhanced CT image obtained after radiotherapy (56 Gy in 28 fractions) showing significant reduction in tumor size (white arrow), with resolution of mass effect on adjacent structures.

Regarding staging, while the World Health Organization (WHO) classification does not define a staging system, the Church Desmoid Staging System [14] classifies it as Stage III. Initial treatment with NSAIDs and tamoxifen resulted in minimal tumor regression. Radiotherapy, delivered at a total dose of 56 Gy in 28 fractions, was subsequently administered, leading to significant tumor reduction (Figure 5). The patient remains under regular follow-up, with imaging studies confirming stable disease over a total course of three years, including two years post-treatment.

## Discussion

DF is locally aggressive but does not metastasize. However, its infiltrative growth and a postoperative recurrence rate of 20%-68% make it a clinical challenge [15]. DF can be classified by location, including extra-abdominal and abdominal fibromatosis. Although lesions can arise in various anatomical sites, they are most commonly found in the extremities (30%-40%), followed by the abdominal wall (20%), retroperitoneum or abdominal cavity (15%), and chest wall (10%-15%) [4,5]. Head and neck involvement, as described in this case, is considerably less frequent, accounting for approximately 6.7% of all DF cases with reported locations, including the submandibular region, posterior neck, and areas surrounding the vertebral column [16]. Among these, tumors around the vertebral column are the most frequently reported [17]. The retropharyngeal and danger spaces, the specific location in our case, represent an exceedingly rare site of origin, often posing significant diagnostic challenges [18].

Histopathologically, DF is characterized by the proliferation of spindle cells arranged in fascicles within a collagen-rich stroma. Excessive collagen production may result in keloid-like collagen and hyalinization. Immunohistochemical staining typically shows strong expression of vimentin and smooth muscle actin (SMA), with nuclear localization of β-catenin observed in up to 80% of cases. Notably, staining for c-kit and CD34 is negative, aiding in differentiation from other spindle cell tumors [1,6].

Imaging plays a critical role in diagnosing DF and distinguishing it from malignant tumors such as undifferentiated pleomorphic sarcoma or synovial sarcoma. In the cervical region, DF typically appears as a spindle-shaped lesion with well-defined margins surrounded by fat [9,17]. CT findings can vary, with DF composed of low-attenuation myxoid components, isodense to mildly hyperdense solid areas, and high-attenuation fibrous regions [15]. Calcification and necrosis are extremely rare. On MRI, DF is typically hyperintense on T2WI and isointense on T1WI. A characteristic feature is the presence of band-like hypointense areas on T2WI, corresponding to dense collagenous stroma, seen in 60%-90% of cases [17]. FDG PET/CT findings demonstrate moderate uptake, with a reported maximum standardized uptake value (SUVmax) of ≤4.7 or a mean of 3.1 (range 2.0-7.3) [9,10]. Some cases also show early contrast staining on angiography [19], extension into the intervertebral foramen [20], and bone remodeling in approximately 20% of cases [9]. Interestingly, a similar case involving the same region, morphology, and characteristics was reported 30 years ago, and that case also demonstrated band-like hypointense signals on T2WI [18]. Among the various findings, this feature may be relatively important for diagnosis.

Many cases of DF remain stable or even regress spontaneously, which is why observation and regular follow-up are often the initial management approach [21]. For progressive or symptomatic tumors, treatment options include surgery, systemic therapy (e.g., NSAIDs, tamoxifen, or tyrosine kinase inhibitors), and radiotherapy [15]. Radiotherapy is particularly effective for unresectable or recurrent cases, with a recommended total dose of 50-60 Gy delivered in 1.8-2.0 Gy fractions [20]. Potential complications of radiotherapy for DF include edema, cellulitis, ulcers, fibrosis, pathological fractures, and secondary malignancies [22].

In this case, DF arose in the retropharyngeal and danger spaces and exhibited rapid growth, complicating differentiation from malignant tumors. Imaging findings, including band-like hypointense areas on T2WI and moderate FDG uptake on PET/CT, played a crucial role in diagnosis. Radiotherapy was administered at a total dose of 56 Gy in 28 fractions, resulting in significant tumor shrinkage and sustained tumor size reduction. No complications were observed during follow-up.

## Conclusions

This case underscores the importance of recognizing the imaging features of DF involving the retropharyngeal and danger space to ensure accurate diagnosis and appropriate management. Although DF is a rare condition, similar imaging findings have been reported, suggesting that careful imaging evaluation can facilitate a preoperative diagnosis, even in atypical locations such as the retropharyngeal and danger spaces. This case highlights the diagnostic challenges posed by DF in such uncommon locations and demonstrates the potential for successful treatment through radiotherapy, which led to significant tumor shrinkage without complications. Further studies are needed to establish optimal treatment protocols for DF in rare locations, and this report contributes valuable insights into the diagnostic and therapeutic approaches for managing rapidly growing DF.
